# EGFR-Targeted Nanoparticle Delivery of Osimertinib for Triple-Negative and Metastatic Breast Cancer Therapy

**DOI:** 10.3390/pharmaceutics18091179

**Published:** 2026-09-18

**Authors:** Iman M. Alfagih, Maryam Alfagih, Alanood Almurshedi, Basmah Aldosari, Bushra Alquadeib, Baraa Hajjar, Shahad Almogheerah, Rund Alzahrani

**Affiliations:** 1 Department of Pharmaceutics, College of Pharmacy, King Saud University, Riyadh 12372, Saudi Arabia; 2 Department of Pharmaceutical Sciences, College of Pharmacy, Alfaisal University, Riyadh 11533, Saudi Arabia

**Keywords:** MCF-7, MDA-MB-231, Osimertinib, chitosan-coated PLGA nanoparticles, breast cancer, triple negative breast cancer

## Abstract

**Background/objective:** Osimertinib, an EGFR-targeted agent approved for metastatic NSCLC, shows promise for triple-negative breast cancer due to EGFR overexpression in aggressive tumors. However, oral administration limits tumor delivery and causes systemic side effects. This study aimed to develop chitosan-coated PLGA nanoparticles loaded with osimertinib (CH-P-NPs) to enhance its anticancer efficacy in breast cancer cell lines (MDA-MB-231, MCF-7) by promoting apoptosis and reducing migration in vitro. **Methods:** CH-P-NPs were synthesized via single-emulsion solvent evaporation and characterized for particle size, zeta potential, encapsulation efficiency, and in vitro release. Anticancer activity was evaluated in MDA-MB-231 and MCF-7 cell lines through in vitro cellular uptake, cytotoxicity, apoptosis induction, and migration inhibition assays. **Results:** CH-P-NPs exhibited a particle size less than 200 nm, high encapsulation efficiency and a positive zeta potential. Moreover, sustained drug release was achieved with 59.62 ± 1.9% at 24 h. In vitro anticancer studies demonstrated that osimertinib and its nanoparticle formulations showed concentration-dependent cytotoxicity in MDA-MB-231 and MCF-7 cells. CH-P-NPs enhanced cytotoxicity compared with uncoated PLGA nanoparticles (IC_50_: 1.56 vs. 6 µg/mL). CH-P-NPs enhanced cellular uptake in MDA-MB-231 cells, whereas its effect on MCF-7 cellular uptake was cell line-dependent. Annexin V/PI assay showed that CH-P-NPs significantly enhanced apoptosis compared to free osimertinib in both cell lines. In MDA-MB-231 cells, CH-P-NPs induced ~52.5% total apoptosis versus 3.3% with free drug. In MCF-7 cells, CH-P-NPs induced ~16.6% apoptosis versus 1.5% with free drug. Late apoptosis predominated, indicating irreversible cell death. Wound healing assay showed that CH-P-NPs significantly inhibited migration in both cell lines compared to free osimertinib and control. In MDA-MB-231 cells, CH-P-NPs exhibited greater inhibition at 48 and 72 h, while free osimertinib showed minimal effect. In MCF-7 cells, CH-P-NPs reduced migration at all time points. **Conclusions:** CH-P-NPs markedly enhanced osimertinib’s anticancer activity by improving cellular uptake, cytotoxicity, inducing apoptosis, and inhibiting migration. This nanoformulation offers a promising strategy to boost efficacy and reduce systemic toxicity in breast cancer treatment.

## 1. Introduction

Triple-negative breast cancer represents an aggressive and heterogeneous subtype of breast cancer, accounting for approximately 10–20% of all cases. Triple-negative breast cancer is highly invasive and tends to metastasize to the brain and visceral organs, particularly the lungs. Clinical data indicate that approximately 46% of patients develop distant metastases within three years of their initial triple-negative breast cancer diagnosis [1,2].

Unlike other forms of breast cancer, triple-negative breast cancer lacks expression of estrogen receptors, progesterone receptors, and human epidermal growth factor receptor 2 (HER2). This receptor deficiency eliminates the possibility of using hormone-based therapies or HER2-targeted treatments, such as tamoxifen or trastuzumab, which are effective in other subtypes. Consequently, treatment options for triple-negative breast cancer are limited, with chemotherapy remaining the primary approach. However, chemotherapy is often associated with high recurrence rates, metastasis, and resistance to drugs. The combination of its aggressive nature and the absence of targeted therapies have made triple-negative breast cancer a critical area of ongoing research aimed at discovering more effective treatment strategies [3].

In 2017, the U.S. Food and Drug Administration granted osimertinib breakthrough therapy status for its role as a first-line treatment in metastatic non-small cell lung cancer harboring EGFR mutations [4]. Similar to non-small cell lung cancer, a subset of triple-negative breast cancer cases has been found to exhibit EGFR mutations [5]. Furthermore, osimertinib is capable of binding to wild-type EGFR [5]. Given that certain aggressive triple-negative breast cancer tumors overexpress EGFR, EGFR-targeted agents such as osimertinib present a promising therapeutic avenue.

Osimertinib is administered orally in the form of a film-coated tablet, which restricts the amount of drug that effectively reaches the tumor site. Its use is often associated with severe systemic side effects, including diarrhea, skin rashes, mucositis, and renal complications, limiting its long-term and widespread application [6,7]. Moreover, patients receiving osimertinib frequently develop acquired resistance, reducing its ability to deliver sustained therapeutic benefits [8,9]. Therefore, creating innovative strategies to overcome the limitations of conventional dosage forms is crucial, as this could address a significant clinical challenge and improve patient survival outcomes.

Nanoparticles are widely recognized as an effective drug delivery system for anticancer agents, offering improved therapeutic efficacy while reducing adverse effects. They can target tumors through both active and passive mechanisms. Passive targeting occurs via the enhanced permeability and retention (EPR) effect, which allows nanoparticles (typically 100–600 nm in size) to accumulate within tumor vasculature. In breast cancer treatment, several nanomedicine formulations have been clinically approved and are routinely used worldwide, including liposomal doxorubicin, albumin-bound paclitaxel, and polyethylene glycol-conjugated L-asparaginase [3].

Chitosan-coated PLGA nanoparticles loaded with anticancer agents are among the most extensively researched nanoparticle-based delivery systems for breast cancer treatment. These formulations have demonstrated significant effectiveness in both in vitro and in vivo studies. For example, Pitchika et al. prepared chitosan-coated PLGA nanoparticles co-loaded with Paclitaxel and Lapatinib, which showed strong synergistic effects, improved drug uptake, and led to significant tumor inhibition in resistant HER2-positive breast cancer [10]. Similarly, Anwer et al. developed chitosan-coated PLGA nanoparticles loaded with Olaparib to improve its low bioavailability. The formulation achieved sustained release and a 4.75-fold increase in bioavailability compared to standard Olaparib, offering a promising strategy for safer, more effective delivery [11].

To date, no studies have explored chitosan-coated PLGA nanoparticles for delivering osimertinib to enhance its anticancer activity in breast cancer cell lines such as MDA-MB-231 and MCF-7. This study aimed to develop osimertinib-loaded chitosan-PLGA nanoparticles to improve therapeutic efficacy by promoting cell apoptosis and reducing migration in vitro. Although MCF-7 cells exhibit relatively low EGFR expression, they were included as a luminal breast cancer model to evaluate the effectiveness of the nanoformulation under conditions of limited EGFR availability and to compare responses across breast cancer subtypes with distinct EGFR profiles.

## 2. Materials and Methods

### 2.1. Materials

Acid-terminated poly(lactic-co-glycolic acid) (PLGA, 50:50; MW 7–17 kDa) was obtained from Liaoning Kuke Biotechnology Co., Ltd. (Liaoning, China). Low-molecular-weight chitosan (MW 50,000–190,000; degree of deacetylation 75–85%) and Fluorescein (free acid) were purchased from Merck KGaA (Darmstadt, Germany). Osimertinib was sourced from Sigma (Shanghai, China). Poly(vinyl alcohol) (PVA, MW 13–23 kDa, 87–89% hydrolyzed), acetonitrile, and dichloromethane were procured from Fisher Scientific (Loughborough, UK). The triple-negative breast cancer cell line MDA-MB-231 (ATCC HTB-26™) and Metastatic Breast Cancer cell line MCF-7 (ATCC HTB-22™) were obtained from the American Type Culture Collection (ATCC, Manassas, VA, USA). Dulbecco’s Modified Eagle medium (DMEM) supplemented with 10% Fetal Bovine serum (FBS) were purchased from ThermoFisher Scientific, Waltham, MA, USA.

### 2.2. Preparation of Nanoparticles

Chitosan-coated PLGA nanoparticles (CH-P NPs) were synthesized by the emulsion solvent evaporation technique [12]. Briefly, 2 mg of osimertinib was dissolved in ethanol and subsequently mixed with an organic phase containing 50 mg of PLGA in dichloromethane. This mixture was subjected to probe sonication (VC X 500 Vibra-CellTM, 13 mm probe, Sonics & Materials, Inc., Newtown, CT, USA) for 120 s at 40% amplitude with a 10 s on/off cycle. The resulting primary emulsion was further sonicated for 240 s under the same condition in an aqueous solution comprising chitosan (1% *w*/*v*) and polyvinyl alcohol (1% *w*/*v*) as a stabilizer. The emulsion was stirred at room temperature 2 h to facilitate solvent evaporation. CH-P-NPs were collected by centrifugation (Sigma 3–30k; SIGMA Laborzentrifugen GmbH, Osterode am Harz, Germany) at 40,000 rcf and 4 °C for 15 min, followed by two washing steps with water under the same centrifugation conditions. Uncoated nanoparticles (P-NPs) were prepared using the same protocol, excluding chitosan from the aqueous phase. Additionally, for cellular uptake studies, fluorescein-loaded NPs were synthesized via the same procedure by substituting osimertinib with fluorescein.

### 2.3. Characterization of Nanoparticles

The samples’ particle size, polydispersity index and zeta potential were analyzed by Zetasizer Nano ZS (Malvern Instruments Limited, Malvern, UK). Measurements were performed at 25 °C after diluting the sample with deionized water, and each analysis was conducted in triplicate (n = 3). The nanoparticles’ morphology was visualized using transmission electron microscope (TEM, JEOL 1400 PLUS, Tokyo, Japan). Samples were placed on formvar/carbon-coated copper grids (200 mesh; Agar Scientific, Rotherham, UK) and air-dried at room temperature for 10 min prior to imaging.

### 2.4. High-Performance Liquid Chromatograph Assay

A Waters high-performance liquid chromatograph system (Waters Corporation, Milford, MA, USA) was used to quantify osimertinib equipped with Waters Symmetry C18 column (4 μm, 3.9 mm × 250 mm). High-performance liquid chromatograph chromatographic conditions were as follows: the mobile phase consisted of 0.1% (*v*/*v*) orthophosphoric acid: acetonitrile at 20:80 (*v*/*v*) ratio at 40 °C. The flow rate was 0.5 mL/min. The detection wavelength was set at 220 nm. The retention time for osimertinib was found to be 1.731 min [13].

The amount of osimertinib encapsulated within the nanoparticles was determined indirectly by quantifying the residual drug present in the supernatant and wash solutions after centrifugation using high-performance liquid chromatography analysis [13]. Collected samples were appropriately diluted with the mobile phase, filtered through a 0.45 μm membrane filter to remove particulate matter, and analyzed under the same chromatographic conditions. The peak areas were measured, and the concentration of free osimertinib was calculated based on a standard calibration curve. Encapsulation efficiency (%EE) was calculated using Equation (1), with all measurements performed in triplicate (n = 3).

(1) $$EE\%=\frac{Amount\;of\;drug\;added-free\;amount\;of\;drug}{Amount\;of\;drug\;added}$$

### 2.5. In Vitro Drug Release

Nanoparticles equivalent to 90 µg of osimertinib were accurately weighed and dispersed in 7.0 mL of phosphate-buffered saline containing 1% polysorbate 80, adjusted to pH 7.4. The suspension was incubated in a water bath at 37 ± 0.5 °C with constant agitation (100 rpm) under light-protected conditions. At predetermined time intervals (1, 2, 4, 6, 8, and 24 h), 0.5 mL of the supernatant was collected by centrifugation, and 120 µL of the clarified supernatant was analyzed using high-performance liquid chromatography. After sampling, nanoparticles were resuspended in fresh medium and incubation continued. Drug release profiles for P-NPs and chitosan-coated CH-P-NPs were expressed as cumulative release percentages.

### 2.6. In Vitro Anti-Cancer Activity

#### 2.6.1. Cell Culture

Breast cancer cell lines (MDA-MB-231 and MCF-7) were cultured in DMEM supplemented with 100 mg/mL of streptomycin, 100 U/mL of penicillin and 10% heat-inactivated fetal bovine serum. Cells were maintained in a humidified incubator at 37 °C under a 5% CO_2_ atmosphere.

#### 2.6.2. Effects on the Viability of MDA-MB-231 and MCF-7 Cells In Vitro

Cell viability was evaluated using the sulforhodamine B (SRB) assay. Briefly, 100 µL of cell suspension (5 × 10^3^ cells/well) was seeded into 96-well plates and incubated in complete medium for 24 h to allow for cell attachment. Subsequently, cells were treated with 100 µL of medium containing the test compounds (free osimertinib, P-NPs and CH-P-NPs) at varying concentrations and incubated for 72 h. After treatment, the medium was replaced with 150 µL of 10% trichloroacetic acid (TCA) to fix the cells, followed by incubation at 4 °C for 1 h. The TCA solution was removed, and wells were rinsed five times with distilled water. Next, 70 µL of SRB solution (0.4% *w*/*v*) was added to each well and incubated in the dark at room temperature for 10 min. Excess dye was removed by washing three times with 1% acetic acid, and plates were air-dried overnight. Finally, 150 µL of 10 mM TRIS buffer was added to solubilize the protein-bound dye, and absorbance was measured at 540 nm using a microplate reader (FLUOstar Omega, BMG LABTECH^®^, Ortenberg, Germany).

#### 2.6.3. Effects on the Cellular Uptake of MDA-MB-231 and MCF-7 Cells In Vitro

Cells were seeded in 6-well plates at a density of 2 × 10^5^ cells/well and incubated overnight. The culture medium was then carefully aspirated to maintain monolayer integrity and replaced with fresh medium containing the NPs for either 8 or 24 h. Following incubation, cells were harvested via trypsinization and resuspended in 1 mL of ice-cold phosphate-buffered saline (PBS). Samples were analyzed using an ACEA Novocyte™ flow cytometer (ACEA Biosciences Inc., San Diego, CA, USA). For each sample, 12,000 events were recorded, and the net fluorescent intensities (NFI) were quantified using ACEA NovoExpress™ version 1.1.0 software.

#### 2.6.4. Effects on the Apoptosis of MDA-MB-231 and MCF-7 Cells In Vitro

Apoptotic and necrotic cell populations were analyzed using an Annexin V-FITC/PI apoptosis detection kit (Abcam Inc., Cambridge, UK) in combination with flow cytometry. Following treatment with the test compounds (free osimertinib and CH-P-NPs) for 72 h, approximately 1 × 10^5^ cells were harvested by trypsinization and rinsed twice with ice-cold phosphate-buffered saline (pH 7.4). Cells were then incubated in the dark with 0.5 mL of Annexin V-FITC/PI staining solution for 30 min at room temperature, following the manufacturer’s protocol. After staining, samples were analyzed using an ACEA NovoCyte™ flow cytometer (ACEA Biosciences Inc., San Diego, CA, USA). Fluorescence signals for FITC and PI were detected using FL1 and FL2 channels, respectively (λ_ex/em: 488/530 nm for FITC and 535/617 nm for PI). For each sample, 12,000 events were recorded, and cell populations positive for FITC and/or PI were quantified by quadrant analysis using ACEA NovoExpress™ version 1.1.0 software.

#### 2.6.5. Effects on the Migration of MDA-MB-231 and MCF-7 Cells In Vitro

Cells were seeded at a density of 2 × 10^5^ cells per well in a pre-coated 12-well plate and allowed to form a confluent monolayer overnight. The following day, a uniform scratch was created across the cell layer using a sterile 200 µL pipette tip, and wells were rinsed thoroughly with phosphate-buffered saline to remove detached cells. Control wells were replenished with fresh medium, while treatment wells received medium containing the test compounds (free osimertinib and CH-P-NPs). Plates were incubated at 37 °C in a humidified atmosphere with 5% CO_2_ between imaging time points. Images of the wound area were captured at specified intervals (0, 24, 48, 72 h) using an inverted microscope (Olympus CKX41, Olympus Corporation, Tokyo, Japan) equipped with phase-contrast optics. Cell migration was quantified by calculating the percentage reduction in the wound area over time according to Equation (2).

(2) $$Wound\;Closure\;\%=\frac{A_{t=0}-A_{t=h}}{A_{t=0}}$$

At = 0 h represents the mean wound area recorded at time zero (immediately post-scratch), whereas At = Δh denotes the mean wound area recorded h hours post-scratch.

### 2.7. Statistical Analysis

Data were analyzed using Minitab^®^ 19 software. One-way ANOVA was applied to assess differences among groups, and Student’s *t*-test was used for pairwise comparisons where appropriate. A *p*-value < 0.05 was considered statistically significant. Results are presented as mean ± standard deviation from three independent experiments.

## 3. Results and Discussion

### 3.1. Characterization of Nanoparticles

As previously noted, osimertinib exhibits poor aqueous solubility and limited bioavailability, which significantly restrict its therapeutic potential against cancer. To address these limitations, osimertinib has been incorporated into various nanoparticle systems to improve its delivery and efficacy [14,15,16]. Chitosan-coated PLGA nanoparticles are widely recognized as effective nanocarriers for enhancing drug stability, bioavailability, and targeted delivery [17,18]. In this study, we developed Osimertinib-loaded CH-P-NPs using the single-emulsion solvent evaporation method, a widely recognized technique for encapsulating hydrophobic drugs within polymeric carriers [19].

**Figure 1:**
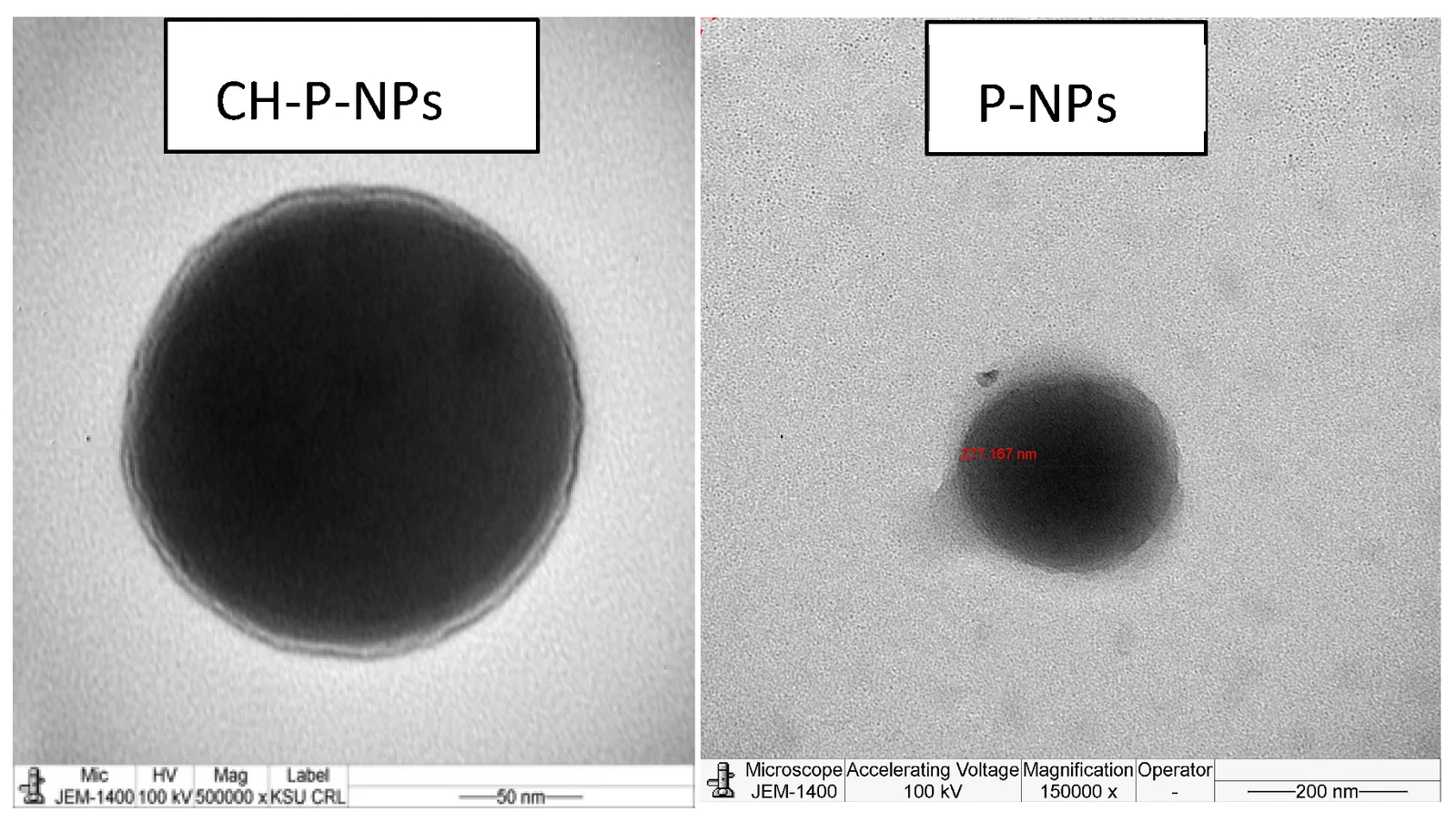
Transmission electron microscopy images: osimertinib-loaded chitosan-coated PLGA nanoparticles (CH-P-NPs) and osimertinib-loaded PLGA nanoparticles (P-NPs).

Zeta potential analysis showed a shift in surface charge from negative to positive, confirming successful chitosan coating on CH-P-NPs (Table 1). The negative charge of P-NPs is attributed to carboxyl end groups, whereas the positive charge of CH-P-NPs results from chitosan’s amino groups on the nanoparticle surface. These findings align with TEM observations, which indicate chitosan forms the outer layer (Figure 1). These results are in agreement with previously reported findings [20,21].

Typically, chitosan coating increases particle size; however, in this study, the size decreased (Table 1), likely due to low-molecular-weight chitosan filling micropores and compressing surface charge [15]. Nanoparticles sized 100–200 nm can penetrate tumor vascular fenestrations via the enhanced permeability and retention (EPR) effect while avoiding rapid clearance by the liver and spleen. In breast cancer, angiogenesis creates leaky, abnormal blood vessels that facilitate passive nanoparticles accumulation through the EPR effect [22].

Both P-NPs and CH-P-NPs exhibited high encapsulation efficiency (Table 1), likely due to osimertinib’s hydrophobic nature, which favors incorporation into the nanoparticle core [23]. The addition of chitosan did not significantly alter EE% (*p* > 0.05), as it was introduced in the aqueous phase and thus had minimal impact on organic phase viscosity or drug diffusion [24]. High EE% is advantageous for reducing material loss, improving yield, and lowering production costs.

### 3.2. In Vitro Release Study

**Figure 2:**
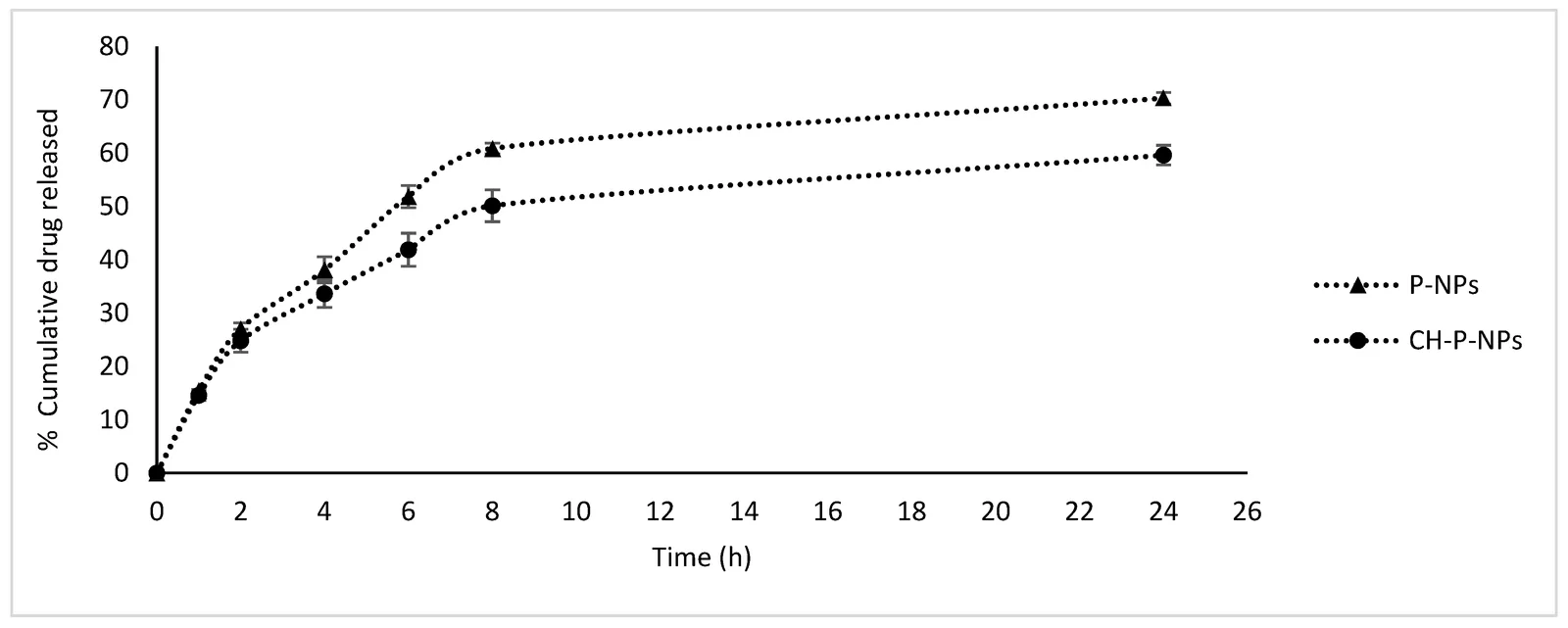
In vitro release of osimertinib-loaded PLGA nanoparticles (P-NPs) and osimertinib-loaded chitosan-coated PLGA nanoparticles (CH-P-NPs) in phosphate-buffered saline with pH 7.4 for 24 h. Data represents mean±SD (n = 3).

To investigate the sustained-release behavior of nanoparticles, cumulative drug release was quantified in phosphate-buffered saline supplemented with 1% polysorbate 80, adjusted to pH 7.4, and maintained at 37 °C (Figure 2). The release profile of osimertinib from nanoparticles exhibited a biphasic pattern. Both P-NPs and CH-P-NPs demonstrated an initial burst release of approximately 24–25% within the first two hours, which can be attributed to the rapid desorption of osimertinib from or near the particle surface, combined with enhanced diffusion of the release medium across the surface. The subsequent slower release phase is likely governed by osimertinib encapsulation within the PLGA polymeric matrix, where diffusion occurs gradually due to the hydrophobic nature of PLGA, which limits water penetration. Furthermore, the extended diffusion pathway of osimertinib molecules within the polymer core and the gradual bioerosion of PLGA contribute to this sustained release. Notably, CH-P-NPs modified with chitosan exhibited a lower release rate compared to P-NPs after four hours, potentially due to the chitosan coating mitigating hydrolytic degradation of the nanoparticle structure [18]. After 24 h, approximately 70.31 ± 1.1% and 59.62 ± 1.9% of osimertinib was released from P-NPs and CH-P-NPs, respectively. This controlled and prolonged release profile is anticipated to enhance therapeutic efficacy by supporting sustained tumor suppression [25].

### 3.3. In Vitro Anti-Cancer Activity

#### 3.3.1. Evaluation of the Cytocompatibility of the Blank Nanoparticles

**Figure 3:**
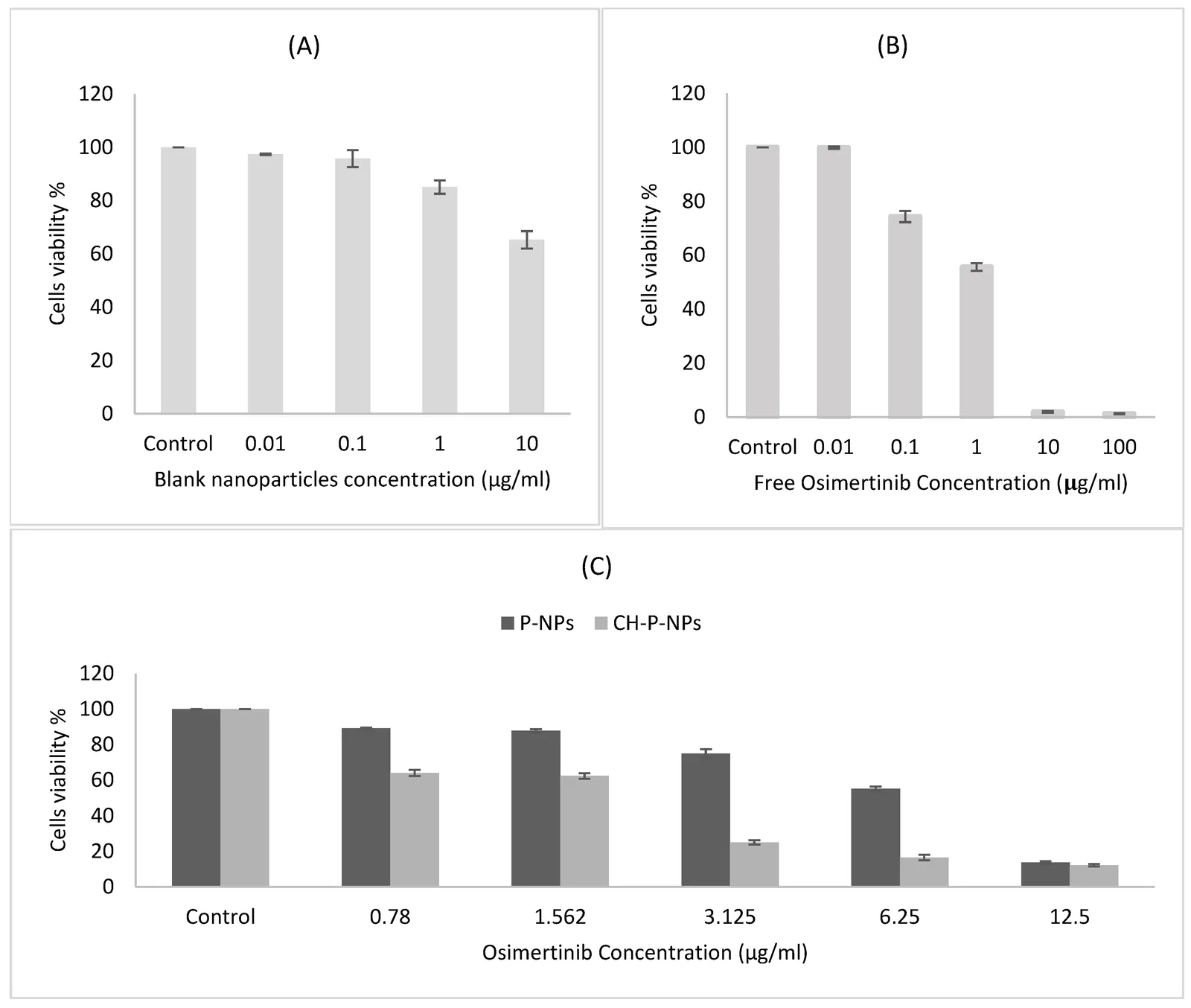
Cytotoxic effects of osimertinib in different formulations on MDA-MB-231 cells were evaluated. Cells were treated for 72 h with (**A**) blank chitosan-coated PLGA nanoparticles, (**B**) varying concentrations of free osimertinib, and (**C**) osimertinib-loaded PLGA nanoparticles (P-NPs) and osimertinib-loaded chitosan-coated PLGA nanoparticles (CH-P-NPs); concentrations refer to osimertinib content. Cell viability was determined using SRB assay. Values are the mean ± SD (n = 3).

**Figure 4:**
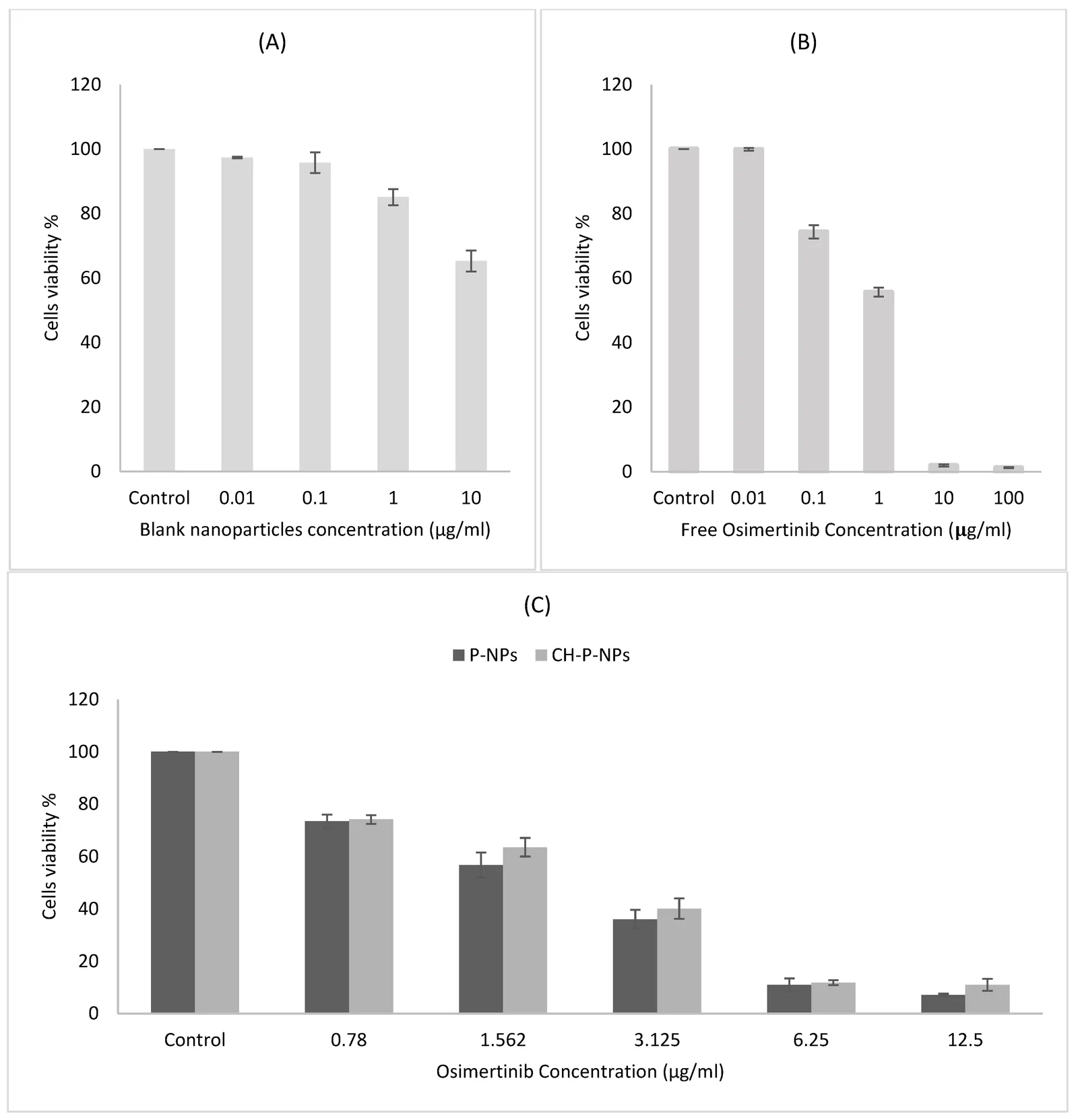
Cytotoxic effects of osimertinib in different formulations on MCF-7 cells were evaluated. Cells were treated for 72 h with (**A**) blank chitosan-coated PLGA nanoparticles, (**B**) varying concentrations of free osimertinib, and (**C**) osimertinib-loaded PLGA nanoparticles (P-NPs) and osimertinib-loaded chitosan-coated PLGA nanoparticles (CH-P-NPs); concentrations refer to osimertinib content. Cell viability was determined using SRB assay. Values are the mean ± SD (n = 3).

The cytocompatibility of the blank nanoparticle formulation was assessed using the SRB assay on MDA-MB-231 and MCF-7 cell lines. As illustrated in Figure 3 and Figure 4, cell viability exhibited only a slight reduction with increasing concentrations of the formulation, remaining above 80% across all tested doses. These results confirm that the blank formulation demonstrates negligible cytotoxicity and excellent biocompatibility, indicating its potential safety for therapeutic applications. Furthermore, the chitosan used in the formulation developed in this study was applied only as a thin surface coating in small amounts, resulting in a moderate positive charge of +21.03 ± 4.50 mV, which lies within the biocompatible range reported for chitosan-based nanoparticles (+15 to +30 mV) [26,27]. Such mild surface modification is considered safe and unlikely to trigger apoptosis or alter cell migration; therefore, the blank CH-P-NPs were not subjected to apoptosis or migration assay.

#### 3.3.2. Effects on the Viability of MCF-7 and MDA-MB-231 Cells In Vitro

The cytotoxic efficacy of free osimertinib, P-NPs and CH-P-NPs against the breast cancer cell lines MDA-MB-231 and MCF-7 was evaluated using the SRB assay. All treatments were assessed after 72 h of exposure. The results indicated a concentration-dependent effect, with cell viability significantly decreasing (*p* < 0.05) as the concentration of each formulation increased (Figure 3B,C and Figure 4B,C).

In the MDA-MB-231 cell line, free osimertinib exhibited an IC_50_ value of 1.05 ± 0.3 µg/mL, indicating its strong potency against MDA-MB-231 cells. Encapsulation of osimertinib within CH-P-NPs produced a comparable cytotoxic effect, with an IC_50_ of 1.56 ± 0.6 µg/mL. It is noteworthy that osimertinib must be released from the nanoparticles before exerting its effect on the cells. This finding aligns with the release study results, which showed that only a 59.62 ± 1.9% of osimertinib was released after 24 h of incubation. CH-P-NPs exhibited an IC_50_ (1.56 ± 0.6 µg/mL) that was approximately 3.85-fold lower compared with P-NPs (IC_50_: 6 ± 1.2 µg/mL; *p* < 0.05). The encapsulation of osimertinib within CH-P-NPs resulted in significantly enhanced therapeutic efficacy at a lower drug dose. This finding aligns with the observation that CH-P-NPs exhibited markedly higher cytotoxicity than P-NPs at equivalent osimertinib concentrations (*p* < 0.05; Figure 3C). The increased cytotoxicity of CH-P-NPs is likely influenced by their positively charged surface, resulting from the chitosan coating. The change from a negatively charged P-NPs to a positively charged CH-P-NPs may enhance electrostatic interactions with the negatively charged cell membrane, which could contribute to their increased inhibitory effect [25]. The encapsulation of anticancer drugs within polymeric or lipid-based nanoparticles further amplifies their therapeutic efficacy. For instance, Mandal et al. synthesized amino acid-based cationic lipids and showed that increasing the positive surface charge, particularly through histidine-rich head groups, significantly enhanced cellular uptake and biological activity in MDA-MB-231 cells [28]. A similar charge-mediated enhancement has been well-documented for chitosan-coated PLGA nanoparticles in MDA-MB-231 cells. For example, Hu et al. demonstrated that chitosan-coated PLGA nanoparticles loaded with thymoquinone significantly enhanced cytotoxicity against MDA-MB-231 breast cancer cells compared to uncoated nanoparticles [29]. Similarly, Payomhom et al. reported that chitosan-coated PLGA nanoparticles loaded with ursolic acid exhibited significantly enhanced cytotoxicity in MDA-MB-231 cells, with a marked reduction in IC_50_ values compared to uncoated nanoparticles, an effect attributed to chitosan-mediated surface charge and improved cellular internalization [20]. This highlights chitosan coating as a promising strategy for improved anticancer drug delivery. Thus, CH-P-NPs were selected for further in vitro anticancer studies due to their enhanced cytotoxicity compared to P-NPs.

In the MCF-7 cell line, free osimertinib exhibited an IC_50_ value of 0.82 ± 0.2 µg/mL, indicating its strong potency against MCF-7 cells. The encapsulation of osimertinib within P-NPs or CH-P-NPs produced a comparable cytotoxic effect, with an IC_50_ of 1.79 ± 0.6 and 2.09 ± 0.7 µg/mL, respectively. This is evident because drug release from these nanoformulations occurs more slowly, resulting in delayed action on the cells compared to free drugs, which are immediately available. Furthermore, nanoparticles offer significant advantages for in vivo systems over in vitro systems by improving drug bioavailability through prolonged circulation, enhancing aqueous solubility, and enabling better targeting of cancer tissues [28].

The divergent responses of the two cell lines to CH-P-NPs can be rationalized based on their intrinsic membrane surface charge properties. Electrokinetic studies have demonstrated that MDA-MB-231 cells possess a significantly higher absolute surface charge density than MCF-7 cells across a wide pH range, notably exhibiting a more negatively charged membrane at physiological pH. This increased negative surface charge arises from a higher density of acidic functional groups, enhanced exposure of anionic phospholipids such as phosphatidylserine, and increased lipid peroxidation [30,31]. These features promote strong electrostatic attraction between the negatively charged cell membrane and positively charged chitosan, facilitating nanoparticle adhesion, membrane interaction, and subsequent cellular uptake. Consequently, in this study, CH-P-NPs achieved significantly superior cytotoxic efficacy in MDA-MB-231 cells compared to uncoated nanoparticles. In contrast, the lower surface charge density and reduced electrostatic activity of MCF-7 membranes diminish the contribution of chitosan-mediated interactions. In this context, the passive diffusion of free osimertinib appears sufficient to achieve high cytotoxicity, while nanoparticle encapsulation introduces additional barriers related to drug release kinetics and intracellular trafficking, resulting in slightly reduced potency. This effect is further explained by the sustained drug release from nanoformulations, which delays cellular exposure compared with the immediate availability of the free drug. Nevertheless, such delayed action in vitro does not diminish the therapeutic relevance of the nanoparticles, as they offer substantial advantages in vivo, including improved aqueous solubility, protection against premature degradation, prolonged systemic circulation, and enhanced tumor accumulation, ultimately supporting superior therapeutic performance under physiological conditions [32].

#### 3.3.3. Effects on the Cellular Uptake of MCF-7 and MDA-MB-231 Cells In Vitro

To determine whether the charge-mediated enhancement in cytotoxicity was directly associated with improved cellular internalization, cellular uptake studies were performed in both MDA-MB-231 and MCF-7 cell lines. Because cellular uptake governs the intracellular accumulation and subsequent therapeutic efficacy of nanoparticles, fluorescein-loaded P-NPs and CH-P-NPs were prepared to quantify internalization via flow cytometry. In this experiment, osimertinib was replaced with fluorescent fluorescein to enable quantification of nanoparticle uptake.

**Figure 5:**
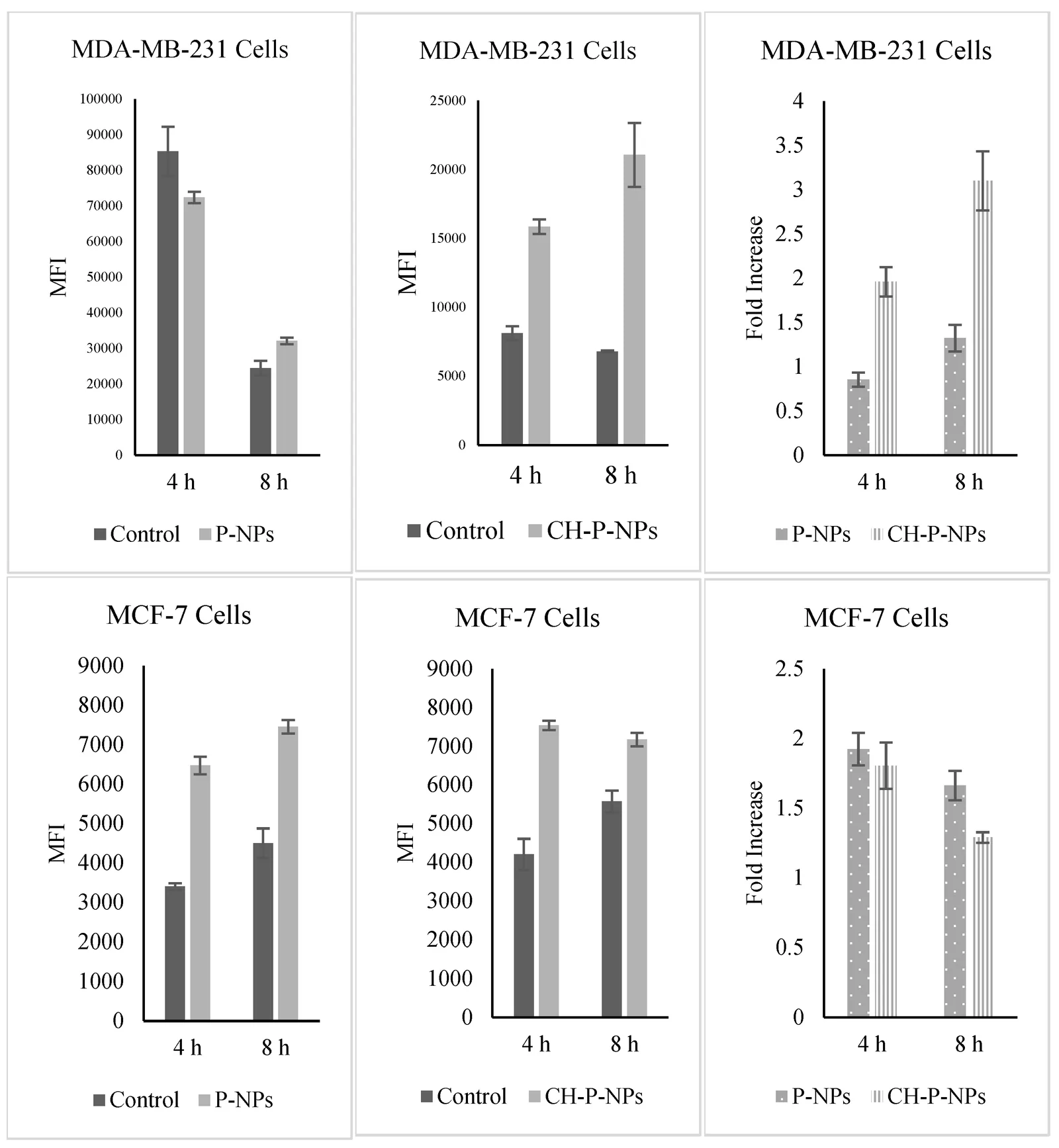
Cellular uptake of PLGA nanoparticles (P-NPs) and chitosan-coated PLGA nanoparticles (CH-P-NPs) by MDA-MB-231 and MCF-7 cells and the corresponding fold increase in uptake relative to untreated control cells. Cells were incubated with fluorescein-loaded P-NPs or CH-P-NPs for 4 and 8 h, and nanoparticle internalization was quantified by flow cytometry. Values are expressed as mean ± SD (n = 3).

In MDA-MB-231 cells (Figure 5), statistical analysis revealed highly significant effects of treatment (*p* < 0.0001), incubation time (*p* < 0.0001), and their interaction (*p* < 0.0001), indicating that the influence of nanoparticle formulation on cellular uptake was dependent on incubation time. Tukey’s post hoc analysis showed that P-NPs at 8 h produced significantly greater uptake than the corresponding control group (*p* = 0.0002), while P-NPs at 4 h exhibited a borderline increase that did not remain significant after multiple-comparison correction (*p* = 0.0555). Relative to their respective controls, CH-P-NPs increased cellular uptake by 1.95-fold at 4 h and 3.10-fold at 8 h, demonstrating a strong time-dependent enhancement in internalization. In contrast, P-NPs showed a 0.85-fold change at 4 h and a 1.31-fold increase at 8 h. Among all experimental groups, the greatest uptake enhancement was observed for CH-P-NPs at 8 h, which achieved a 3.10-fold increase over the corresponding control, highlighting the superior uptake efficiency of the coated nanoparticle formulation in MDA-MB-231 cells.

In MCF-7 cells (Figure 5), statistical analysis demonstrated significant effects of both nanoparticle formulation and incubation time on cellular uptake in MCF-7 cells. A highly significant main effect of treatment was observed (*p* < 0.0001), together with a significant effect of incubation time (*p* < 0.0001), whereas the treatment × time interaction was not significant (*p* = 0.500). Tukey’s multiple-comparison test confirmed that both P-NPs and CH-P-NPs formulations exhibited significantly greater uptake than the corresponding control groups. P-NPs increased cellular uptake by 1.92-fold and 1.66-fold after 4 h and 8 h of incubation, respectively, whereas CH-P-NPs enhanced uptake by 1.79-fold and 1.29-fold after 4 h and 8 h, respectively.

The differential uptake observed between MDA-MB-231 and MCF-7 cells suggests that the influence of chitosan coating is highly cell line-dependent. Due to the presence of cationic amino groups, chitosan can enhance nanoparticle interactions with negatively charged cell membranes and facilitate endocytic internalization through mechanisms such as clathrin-mediated and caveolae-mediated endocytosis, depending on both nanoparticle characteristics and cell line type [33]. In the present study, CH-P-NP uptake in MDA-MB-231 cells increased markedly over time, rising from 1.95-fold at 4 h to 3.10-fold at 8 h, indicating a pronounced time-dependent enhancement of nanoparticle internalization compared with P-NPs. In contrast, MCF-7 cells exhibited relatively higher uptake at 4 h followed by a decline at 8 h for both P-NPs and CH-P-NPs. This pattern is consistent with previous reports demonstrating differences in endocytic activity and intracellular transport pathways between MDA-MB-231 and MCF-7 cells, which may underlie their distinct responses to nanoparticle surface modification [20,34]. Voronovic et al. reported that MDA-MB-231 cells are enriched in proteins involved in cellular transport and endocytosis, including components of clathrin-coated vesicles, whereas MCF-7 cells show greater enrichment of proteins associated with cytoskeletal organization and cell–cell adhesion. Cell morphology may also contribute to these differences, as MCF-7 cells typically grow as compact epithelial-like colonies with strong intercellular contacts, whereas MDA-MB-231 cells display a more dispersed and invasive phenotype. Such characteristics can influence the extent of plasma membrane exposure to nanoparticles, thereby affecting nanoparticle binding and subsequent internalization [34]. These findings are further supported by the study of Payomhom et al., who investigated the cellular uptake of PLGA/chitosan nanoparticles in MCF-7 and MDA-MB-231 cells and demonstrated a clear time- and dose-dependent internalization pattern in both cell lines, with significantly higher uptake in MDA-MB-231 cells than in MCF-7 cells at equivalent nanoparticle concentrations. The authors also emphasized that cellular uptake behavior is strongly influenced by nanoparticle composition and surface properties, noting that previous studies using uncoated PLGA nanoparticles reported the opposite uptake pattern [20]. For a more detailed evaluation of the differential uptake of PLGA/chitosan nanoparticles between MDA-MB-231 and MCF-7 cells and the underlying mechanisms of cellular internalization, readers are referred to the study by Payomhom et al. [20]. Collectively, these observations highlight the critical role of nanoparticle surface modification in governing cellular interactions.

#### 3.3.4. Effects on the Apoptosis of MCF-7 and MDA-MB-231 Cells In Vitro

**Figure 6:**
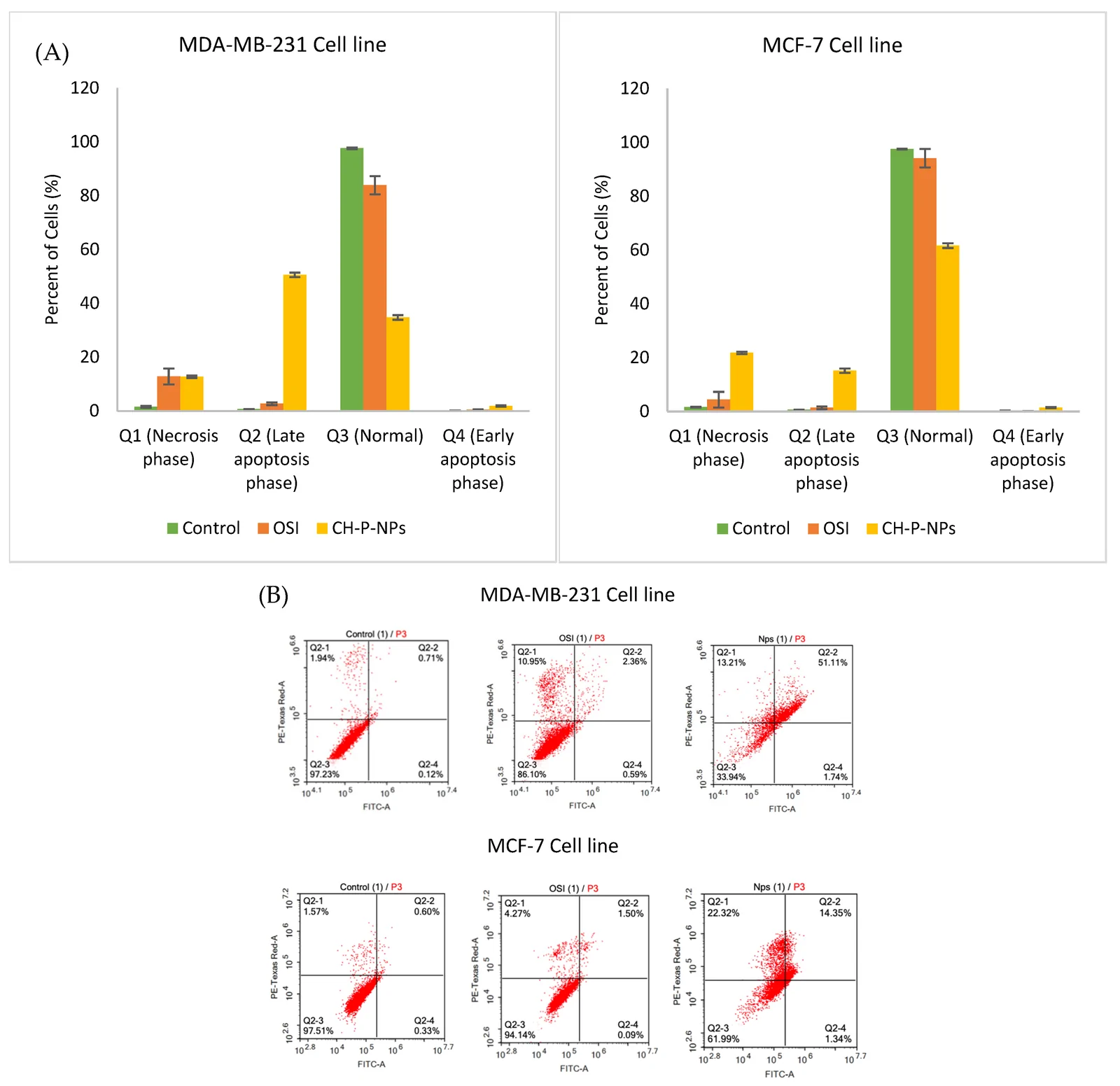
Apoptosis analysis in MDA-MB-231 and MCF-7 cells after 72 h treatment with control (untreated), free osimertinib (OSI), and osimertinib-loaded chitosan-coated PLGA nanoparticles (CH-P-NPs). Apoptosis was assessed using Annexin V-FITC/PI staining and flow cytometry. (**A**) Bar graph showing the quantitative distribution of cell populations (live, early apoptotic, late apoptotic, and necrotic) for each treatment group. (**B**) Representative flow cytometry dot plots illustrating the quadrant-based classification of cell populations for the same treatments.

To further investigate the impact of nanoparticles on cell viability, an Annexin V/PI apoptosis assay was conducted. Apoptosis is a programmed cell death mechanism that eliminates cells with severe DNA damage, preventing deleterious effects such as tumorigenesis [28]. Since evasion of apoptosis is a hallmark of cancer, inducing apoptosis in malignant cells represents a key therapeutic strategy [29]. Annexin V (AV), a Ca^2+^-dependent phospholipid-binding protein, exhibits high affinity for phosphatidylserine (PS), which is normally located on the inner leaflet of the plasma membrane. Following Annexin V-FITC/PI staining, four cell populations can be distinguished: live, necrotic, early apoptotic, and late apoptotic cells [28]. Annexin V/PI staining revealed distinct apoptotic profiles in both cell lines following treatment (Figure 6A,B).

In the MDA-MB-231 cell line, the control group exhibited 0.18 ± 0.06% early apoptotic cells and 0.67 ± 0.05% late apoptotic cells. Treatment with free osimertinib resulted in 0.54 ± 0.04% early apoptosis and 2.72 ± 0.51% late apoptosis. In contrast, CH-P-NPs treatment led to a marked increase in apoptosis, with 1.9 ± 0.2% early apoptotic cells and 50.6 ± 0.8% late apoptotic cells. The total apoptotic population in the nanoparticle group reached approximately 52.5%, which was significantly higher than that observed with free osimertinib (3.26%) (*p* < 0.05). Similarly, in the MCF-7 cell line, the control group exhibited 0.20 ± 0.08% early apoptotic cells and 0.60 ± 0.05% late apoptotic cells. Treatment with free osimertinib resulted in 0.12 ± 0.03% early apoptosis and 1.34 ± 0.18% late apoptosis. In contrast, CH-P-NPs treatment significantly increased apoptosis, with 1.4 ± 0.1% early apoptotic cells and 15 ± 0.16% late apoptotic cells (*p* < 0.05). The total apoptotic population in the nanoparticle group reached approximately 16.6%, indicating that CH-P-NPs effectively induce apoptosis in MCF-7 cells. Notably, the substantially higher apoptotic response observed in MDA-MB-231 cells compared with MCF-7 cells is consistent with the cytotoxicity results, which demonstrated that CH-P-NPs exert greater cytotoxic effects in MDA-MB-231 cells than in MCF-7 cells.

Collectively, these findings indicate that CH-P-NPs bias cells toward a more advanced apoptotic phenotype compared with free osimertinib in both cell lines, as evidenced by the increased proportion of late apoptotic cells (Figure 6A,B). This suggests enhanced commitment to irreversible cell death despite comparable overall cytotoxicity. This is consistent with previous studies showing that osimertinib nanoformulations can modulate the mode, timing, and intracellular fate of drug-induced cell death without necessarily producing proportionally greater short-term viability loss. For instance, chitooligosaccharide-modified PLGA nanoparticles have been shown to enhance intracellular drug accumulation and sustain intracellular exposure, leading to amplified activation of apoptotic pathways (e.g., caspase activation and PARP cleavage) even when bulk cytotoxicity remains comparable to, or lower than, that of free drug due to rapid passive diffusion in vitro [15]. Similarly, perfluorocarbon-based nanoemulsions of osimertinib have been reported to exhibit delayed but more apoptotically committed cell death, highlighting that free osimertinib can produce equivalent or greater immediate cytotoxicity while nano-encapsulated formulations bias cells toward programmed apoptosis rather than mixed cytostatic or non-apoptotic outcomes [14]. Thus, CH-P-NPs represent effective pro-apoptotic carriers for osimertinib. Future studies will focus on further optimization of PLGA polymer properties and chitosan coating parameters to enhance sustained intracellular drug delivery and apoptotic commitment. In addition, mechanistic investigations involving detailed apoptotic signaling analyses are warranted to better define the therapeutic potential and durability of response of this nanoformulation.

#### 3.3.5. Evaluation on Cell Migration of MCF-7 and MDA-MB-231 Cells In Vitro

Metastasis, particularly to the lungs, brain, and bones, is one of the most defining characteristics of triple-negative breast cancer compared to other breast cancer subtypes. Evidence from multiple studies indicates that nearly 36% of triple-negative breast cancer cases develop metastatic lesions in the central nervous system [35]. In this study, we investigated the antimigratory effects of osimertinib before and after its encapsulation into CH-P-NPs using two breast cancer cell lines: MDA-MB-231 cells, known for their highly aggressive, metastatic, and invasive nature, and MCF-7 cells. The wound healing assay, a widely used in vitro migration model, was employed to evaluate cell motility. This assay involves creating a gap in a confluent monolayer and monitoring the extent of cell migration into the gap over time. To minimize cytotoxic interference, experiments were conducted at concentrations below the IC_50_ of free osimertinib and CH-P-NPs. The primary objective was to determine whether CH-P-NPs could more effectively inhibit the migration of these cancer cells compared to free osimertinib.

**Figure 7:**
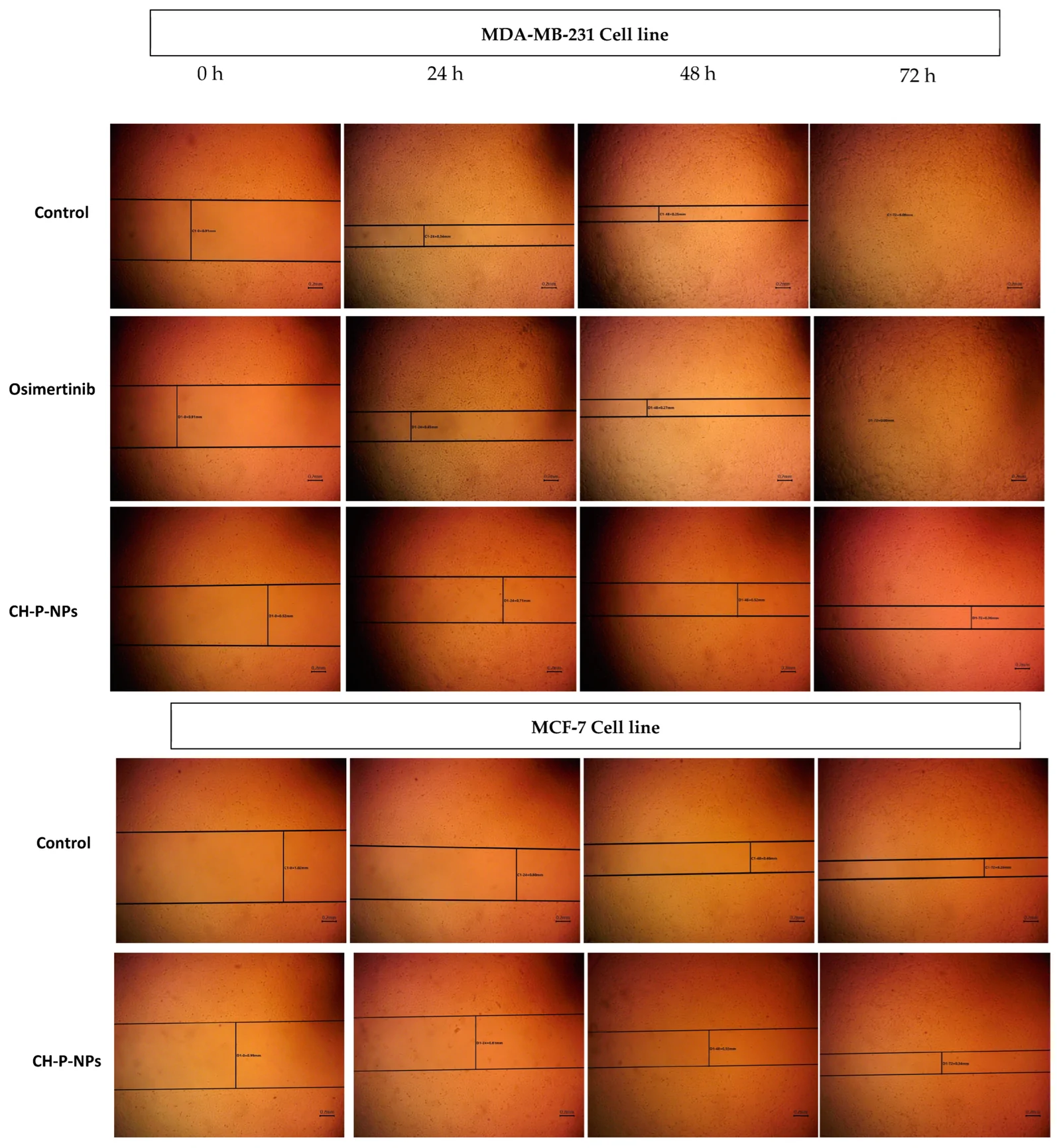
Wound healing microscopic images of the wound at 0 h, 24 h, 48 h and 72 h after treatment with normal medium (control), osimertinib (OSI), or chitosan-coated PLGA nanoparticles (CH-P-NPs) for 72 h. Scale bars = 200 μm.

**Figure 8:**
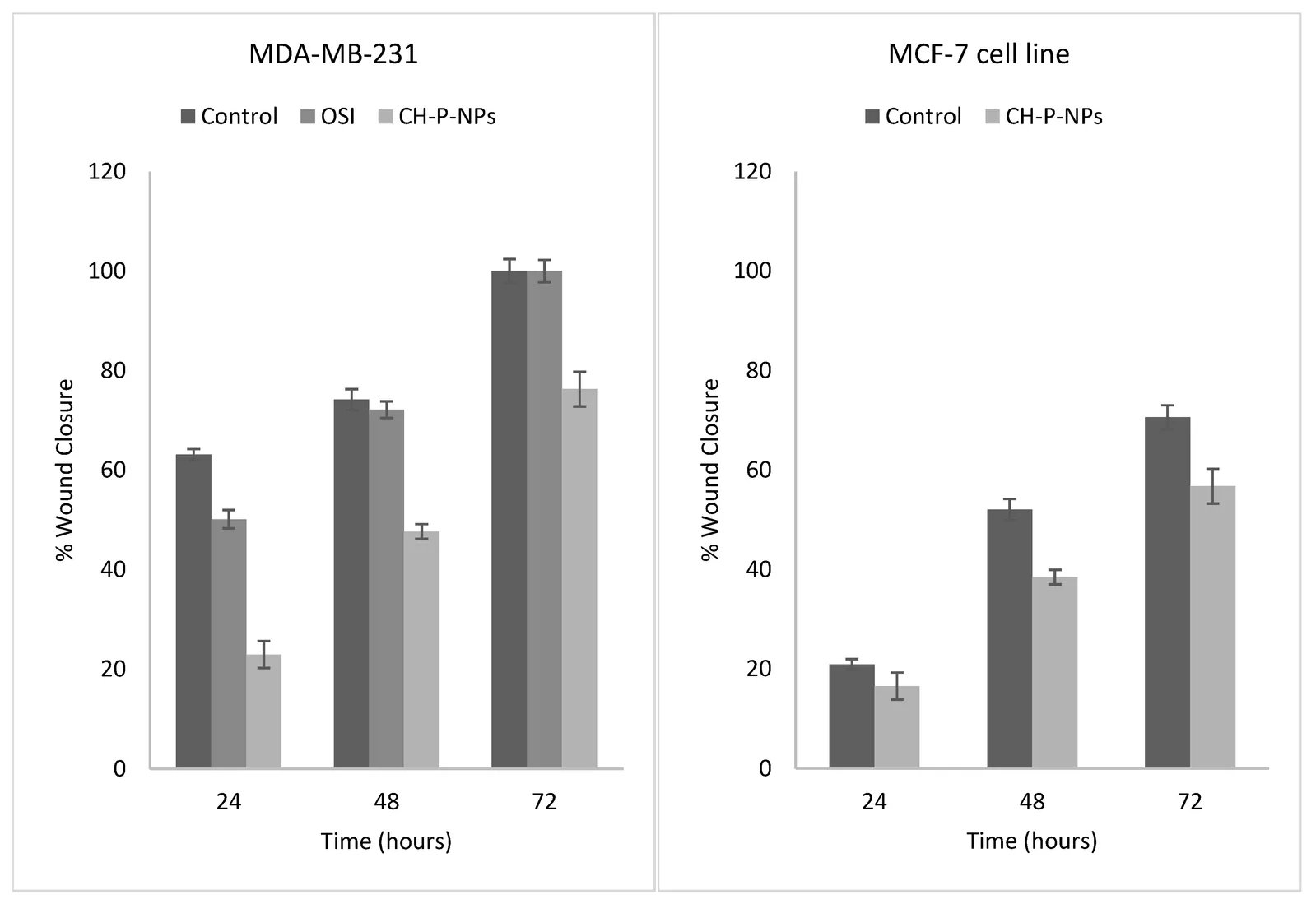
Quantitative assessment of wound closure in breast cancer cells treated with normal medium (control), osimertinib (OSI), or chitosan-coated PLGA nanoparticles (CH-P-NPs) for 72 h. Results are expressed as percentage wound closure over time. Data are presented as mean ± SD (n = 3).

In MDA-MB-231 cells, both free osimertinib and CH-P-NPs exhibited a significant antimigratory effect after 24 h compared to the control group (*p* < 0.05) (Figure 7 and Figure 8). At 48 and 72 h, CH-P-NPs induced a markedly greater inhibition of cell migration compared to both free osimertinib and the control group (*p* < 0.05), whereas no significant difference was observed between free osimertinib and the control group (*p* > 0.05). These findings are consistent with previous reports [17,20,36]. For example, Fong et al. demonstrated that Stattic encapsulated within a chitosan-PLGA nanocarrier exhibited superior in vitro antimigratory activity on MDA-MB-231 cells compared to free Stattic by 65.1% [17]. Similarly, Payomhom et al. reported that ursolic acid-loaded PLGA/chitosan nanoparticles significantly reduced MDA-MB-231 cell migration and invasion compared to free ursolic acid [20]. In MCF-7 cells, CH-P-NPs exhibited a significant antimigratory effect at all tested time points (24, 48, and 72 h) compared to the control group (*p* < 0.05). Furthermore, MDA-MB-231 cells were selected as the primary focus of this study because they represent the highly aggressive triple-negative breast cancer subtype and demonstrated greater sensitivity to osimertinib in the cytotoxicity studies than MCF-7 cells. Therefore, the antimigratory activity of free osimertinib was evaluated only in MDA-MB-231 cells, whereas the effects of the optimized CH-P-NP formulation were assessed in both cell lines.

In comparing the effects of CH-P-NPs on both cell lines, MCF-7 cells exhibited a significantly greater response than MDA-MB-231 cells (*p* < 0.05) (Figure 7 and Figure 8). This difference can be attributed to intrinsic biological and phenotypic distinctions between the two breast cancer models. MCF-7 cells represent a luminal, estrogen receptor-positive, noninvasive phenotype and are well known to exhibit low basal migratory capacity under standard conditions. They maintain strong cell–cell adhesion and higher E cadherin expression and rely on tightly regulated epithelial migration mechanisms. In contrast, MDA-MB-231 cells are triple-negative, highly aggressive, and display a mesenchymal phenotype characterized by elevated basal motility, epithelial–mesenchymal transition (EMT)-associated signaling, enhanced actin remodeling, and increased expression of matrix-degrading enzymes [37]. These intrinsic differences are evident from the in vitro cell migration assays performed in MCF-7 and MDA-MB-231 cells. In the untreated control groups, MDA-MB-231 cells achieved complete wound closure after 72 h, whereas MCF-7 cells exhibited only 70.61 ± 2.4% closure over the same period (Figure 7 and Figure 8). Following treatment with CH -P-NPs, wound closure after 72 h was reduced to 56.75 ± 3.5% in MCF-7 cells and 76.3 ± 3.1% in MDA-MB-231 cells, confirming a significantly greater inhibition of migration in the MCF-7 cell line. Consequently, treatments that interfere with cytoskeletal organization, cell adhesion, or migration-associated signaling pathways may produce a more pronounced relative inhibitory effect in MCF-7 cells, whose migration depends on more tightly regulated epithelial mechanisms [38]. By contrast, the intrinsically high motility and mesenchymal plasticity of MDA-MB-231 cells may enable compensatory migration pathways, thereby attenuate the observable anti-migratory response, and require stronger or more prolonged intervention to achieve comparable inhibition [38,39]. Additionally, differences in cell–nanoparticle interactions may contribute to the observed effects. The epithelial nature of MCF-7 cells, characterized by tighter membrane organization, may favor surface retention and local accumulation of nanoparticles, enhancing interference with migration-related signaling [40]. Conversely, the aggressive migratory machinery of MDA-MB-231 cells may partially compensate for such inhibitory effects, resulting in a reduced overall response [40]. Importantly, the greater inhibition observed in MCF-7 cells does not imply superior therapeutic susceptibility but rather reflects phenotype-dependent baseline migratory behavior. Notably, the significant reduction in migration observed in MDA-MB-231 cells despite their highly invasive nature underscores the robustness and broad anti-migratory potential of the CH-P-NPs’ formulation.

## 4. Conclusions

This study demonstrates that CH-P-NPs represent a promising nanoparticulate delivery system for osimertinib, particularly for enhancing its in vitro anticancer activity against the aggressive MDA-MB-231 breast cancer cell line. The developed formulation exhibited favorable physicochemical characteristics and sustained drug release, together with enhanced cytotoxicity, cellular uptake, apoptosis induction, and inhibition of cell migration compared with free osimertinib. CH-P-NPs also demonstrated anticancer activity against MCF-7 cells; however, their effects were less pronounced than those observed in MDA-MB-231 cells, particularly with respect to apoptosis induction and inhibition of cell migration. These findings suggest that the therapeutic response to CH-P-NPs is dependent on the biological characteristics of the target breast cancer cells. The cellular uptake findings further indicate that the contribution of the chitosan coating is cell-dependent, with a more evident uptake advantage in MDA-MB-231 cells, whereas uncoated P-NPs showed greater uptake in MCF-7 cells. Therefore, chitosan should not be considered universally necessary for nanoparticle-mediated delivery but may provide specific advantages depending on the target cell type. Overall, the findings support the potential of CH-P-NPs to improve the in vitro anticancer performance of osimertinib, particularly against aggressive breast cancer cells. Further studies, including direct evaluation of P-NPs in apoptosis assays and in vivo investigations, are warranted to clarify the specific contribution of chitosan and to validate the therapeutic potential of the developed formulation.

## Figures and Tables

**Table 1:** Particle size and zeta potential of osimertinib-loaded chitosan-coated PLGA nanoparticles (CH-P-NPs) and osimertinib-loaded PLGA nanoparticles (P-NPs). Data represents mean ± SD (n = 3).

Formulation	Particle Size (nm)	Zeta Potential (mV)	Encapsulation Efficiency (%)
P-NPs	202.19 ± 74.5	−18.80 ± 0.60	97.20 ± 0.10
CH-P-NPs	156.20 ± 24.7	+21.03 ± 4.50	96.01 ± 0.40

## Data Availability

The raw data supporting the conclusions of this article will be made available by the authors on request.

## References

[1] You K.S., Yi Y.W., Cho J., Park J.S., Seong Y.S. (2021). Potentiating therapeutic effects of epidermal growth factor receptor inhibition in triple-negative breast cancer. Pharmaceuticals.

[2] Yin L., Duan J.J., Bian X.W., Yu S.C. (2020). Triple-negative breast cancer molecular subtyping and treatment progress. Breast Cancer Res..

[3] Chaudhuri A., Kumar D.N., Dehari D., Singh S., Kumar P., Bolla P.K., Kumar D., Agrawal A.K. (2022). Emergence of Nanotechnology as a Powerful Cavalry against Triple-Negative Breast Cancer (TNBC). Pharmaceuticals.

[4] Odogwu L., Mathieu L., Goldberg K.B., Blumenthal G.M., Larkins E., Fiero M.H., Rodriguez L., Bijwaard K., Lee E.Y., Philip R. (2018). FDA Benefit-Risk Assessment of Osimertinib for the Treatment of Metastatic Non-Small Cell Lung Cancer Harboring Epidermal Growth Factor Receptor T790M Mutation. Oncologist.

[5] Veccia A., Dipasquale M., Lorenzi M., Monteverdi S., Kinspergher S., Zambotti E., Caffo O. (2025). Osimertinib in the Treatment of Epidermal Growth Factor Receptor-Mutant Early and Locally Advanced Stages of Non-Small-Cell Lung Cancer: Current Evidence and Future Perspectives. Cancers.

[6] Lai Y., Zhang Z., Li J., Sun D., Zhou Y.A., Jiang T., Han Y., Huang L., Zhu Y., Li X. (2013). EGFR Mutations in Surgically Resected Fresh Specimens from 697 Consecutive Chinese Patients with Non-Small Cell Lung Cancer and Their Relationships with Clinical Features. Int. J. Mol. Sci..

[7] Díaz-Serrano A., Gella P., Jiménez E., Zugazagoitia J., Paz-Ares Rodríguez L. (2018). Targeting EGFR in Lung Cancer: Current Standards and Developments. Drugs.

[8] Jelinek M.J., Aggarwal C. (2021). Adjuvant Osimertinib: A New Standard of Care. Oncologist.

[9] Bartholomew C., Eastlake L., Dunn P., Yiannakis D. (2017). EGFR targeted therapy in lung cancer; an evolving story. Respir. Med. Case Rep..

[10] Pitchika S., Sahoo S.K. (2022). Paclitaxel and Lapatinib dual loaded chitosan-coated PLGA nanoparticles enhance cytotoxicity by circumventing MDR1-mediated trastuzumab resistance in HER2 positive breast cancers: In-vitro and in-vivo studies. J. Drug Deliv. Sci. Technol..

[11] Anwer M.K., Ali E.A., Iqbal M., Ahmed M.M., Aldawsari M.F., Saqr A.A., Alalaiwe A., Soliman G.A. (2022). Development of Chitosan-Coated PLGA-Based Nanoparticles for Improved Oral Olaparib Delivery: In Vitro Characterization, and In Vivo Pharmacokinetic Studies. Processes.

[12] Raval M., Patel P., Airao V., Bhatt V., Sheth N. (2021). Novel Silibinin Loaded Chitosan-Coated PLGA/PCL Nanoparticles Based Inhalation Formulations with Improved Cytotoxicity and Bioavailability for Lung Cancer. Bionanoscience.

[13] Alhabardi S., Aljohar H., Alganamy A.M., Almurshedi A.S. (2025). A fast, sensitive and greener stability-indicating HPLC method for the quantitative determination of osimertinib. Acta Chromatogr..

[14] Yang J., Li Y., Sun J., Zou H., Sun Y., Luo J., Xie Q., A R., Wang H., Li X. (2022). An Osimertinib-Perfluorocarbon Nanoemulsion with Excellent Targeted Therapeutic Efficacy in Non-small Cell Lung Cancer: Achieving Intratracheal and Intravenous Administration. ACS Nano.

[15] Hu X., Chen S., Yin H., Wang Q., Duan Y., Jiang L., Zhao L. (2020). Chitooligosaccharides-modified PLGA nanoparticles enhance the antitumor efficacy of AZD9291 (Osimertinib) by promoting apoptosis. Int. J. Biol. Macromol..

[16] Sawant S.S., Patil S.M., Shukla S.K., Kulkarni N.S., Gupta V., Kunda N.K. (2022). Pulmonary delivery of osimertinib liposomes for non-small cell lung cancer treatment: Formulation development and in vitro evaluation. Drug Deliv. Transl. Res..

[17] Fong S.S., Foo Y.Y., Saw W.S., Leo B.F., Teo Y.Y., Chung I., Goh B.T., Misran M., Imae T., Chang C.C. (2022). Chitosan-Coated-PLGA Nanoparticles Enhance the Antitumor and Antimigration Activity of Stattic—A STAT3 Dimerization Blocker. Int. J. Nanomed..

[18] Alshehri S., Imam S.S., Rizwanullah M., Fakhri K.U., Rizvi M.M., Mahdi W., Kazi M. (2021). Effect of Chitosan Coating on PLGA Nanoparticles for Oral Delivery of Thymoquinone: In Vitro, Ex Vivo, and Cancer Cell Line Assessments. Coatings.

[19] Nava-Arzaluz M.G., Pinon-Segundo E., Ganem-Rondero A., Lechuga-Ballesteros D. (2012). Single emulsion-solvent evaporation technique and modifications for the preparation of pharmaceutical polymeric nanoparticles. Recent Pat. Drug Deliv. Formul..

[20] Payomhom P., Panyain N., Sakonsinsiri C., Wongtrakoongate P., Lertsuwan K., Pissuwan D., Katewongsa K.P. (2024). Chitosan-Coated Poly(lactic-co-glycolic acid) Nanoparticles Loaded with Ursolic Acid for Breast Cancer TherapY. ACS Appl. Nano Mater..

[21] Alshememry A., Kalam M.A., Almoghrabi A., Alzahrani A., Shahid M., Khan A.A., Haque A., Ali R., Alkholief M., Binkhathlan Z. (2022). Chitosan-coated poly (lactic-co-glycolide) nanoparticles for dual delivery of doxorubicin and naringin against MCF-7 cells. J. Drug Deliv. Sci. Technol..

[22] Blanco E., Shen H., Ferrari M. (2015). Principles of nanoparticle design for overcoming biological barriers to drug delivery. Nat. Biotechnol..

[23] Liang X., Liu S., Deng L., Liu W., Jiang Y. (2025). Study on the formation mechanism and effective manipulation of polymorphs and solvates in Osimertinib-Caffeic acid multi-component crystal with distinct properties. Int. J. Pharm..

[24] Elbatanony R.S., Parvathaneni V., Kulkarni N.S., Shukla S.K., Chauhan G., Kunda N.K., Gupta V. (2021). Afatinib-loaded inhalable PLGA nanoparticles for localized therapy of non-small cell lung cancer (NSCLC)-development and in-vitro efficacy. Drug Deliv. Transl. Res..

[25] Alhakamy N.A., Shadab M. (2019). Repurposing Itraconazole Loaded PLGA Nanoparticles for Improved Antitumor Efficacy in Non-Small Cell Lung Cancers. Pharmaceutics.

[26] Aibani N., Rai R., Patel P., Cuddihy G., Wasan E.K. (2021). Chitosan Nanoparticles at the Biological Interface: Implications for Drug Delivery. Pharmaceutics.

[27] Ezzaki C., Chaari A., Al-Othman A. (2025). Recent Advances on Chitosan-Based Nanoparticles for Brain Drug Delivery. Polymers.

[28] Kalyane D., Raval N., Maheshwari R., Tambe V., Kalia K., Tekade R.K. (2019). Employment of enhanced permeability and retention effect (EPR): Nanoparticle-based precision tools for targeting of therapeutic and diagnostic agent in cancer. Mater. Sci. Eng. C.

[29] Gao J., Kumari A., Zeng X.A., Chan S., Farooq M.A., Alee M., Khan S.H., Rahaman A., He S., Xin X. (2023). Coating of chitosan on poly D,L-lactic-co-glycolic acid thymoquinone nanoparticles enhances the anti-tumor activity in triple-negative breast cancer. Front. Chem..

[30] Dobrzyńska I., Skrzydlewska E., Figaszewski Z.A. (2013). Changes in electric properties of human breast cancer cells. J. Membr. Biol..

[31] Nohara K., Wang F., Spiegel S. (1998). Glycosphingolipid composition of MDA-MB-231 and MCF-7 human breast cancer cell lines. Breast Cancer Res. Treat..

[32] Khan M., Ferdaus J., Akter K., Ahmed H., Parvin M., Kashif S., Arbab A.S. (2025). A comprehensive review of cancer drug nanoparticles synthesis, processing technology and its effect in drug delivery. Biomed. Technol..

[33] Behzadi S., Serpooshan V., Tao W., Hamaly M.A., Alkawareek M.Y., Dreaden E.C., Brown D., Alkilany A.M., Farokhzad O.C., Mahmoudi M. (2017). Cellular Uptake of Nanoparticles: Journey Inside the Cell. Chem. Soc. Rev..

[34] Voronovic E., Voronovic E., Skripka A., Jarockyte G., Ger M., Kuciauskas D., Kaupinis A., Valius M., Rotomskis R., Vetrone F. (2021). Uptake of Upconverting Nanoparticles by Breast Cancer Cells: Surface Coating versus the Protein Corona. ACS Appl. Mater. Interfaces.

[35] Zimmer A.S. (2021). Triple-negative breast cancer central nervous system metastases from the laboratory to the clinic. Cancer J..

[36] Alfagih I.M., Almurshedi A., Aldosari B., Alquadeib B., Hajjar B., Elwali H., ALtukhaim H., Alzahrani E., Alhumaidan S., Alharbi G. (2026). Pulmonary Delivery of Inhalable Sustained Release Nanocomposites Microparticles Encapsulating Osimertinib for Non-Small Cell Lung Cancer Therapy. Pharmaceutics.

[37] Moon H.R., Ospina-Muñoz N., Noe-Kim V., Yang Y., Elzey B.D., Konieczny S.F., Han B. (2020). Subtype-specific characterization of breast cancer invasion using a microfluidic tumor platform. PLoS ONE.

[38] Ouyang M., Chen W., Zhou T., Liu H., Liu L., Bu B., Deng L. (2025). The underlying difference of metastatic and non-metastatic breast cancer cells in configuring type I collagen fibres to promote migration by cell mechanics. Mechanobiol. Med..

[39] Xu X., Xu S., Wan J., Wang D., Pang X., Gao Y., Ni N., Chen D., Sun X. (2023). Disturbing cytoskeleton by engineered nanomaterials for enhanced cancer therapeutics. Bioact. Mater..

[40] Austria E., Bilek M., Varamini P., Akhavan B. (2025). Breaking biological barriers: Engineering polymeric nanoparticles for cancer therapy. Nano Today.

